# Rational combination treatment with histone deacetylase inhibitors and immunomodulatory drugs in multiple myeloma

**DOI:** 10.1038/bcj.2015.38

**Published:** 2015-05-15

**Authors:** T Hideshima, F Cottini, H Ohguchi, J Jakubikova, G Gorgun, N Mimura, Y-T Tai, N C Munshi, P G Richardson, K C Anderson

**Affiliations:** 1 Department of Medical Oncology, Dana-Farber Cancer Institute and Harvard Medical School, Boston, MA, USA

## Abstract

Immunomodulatory drugs (IMiDs) thalidomide, lenalidomide (Len) and pomalidomide trigger anti-tumor activities in multiple myeloma (MM) by targetting cereblon and thereby impacting IZF1/3, c-Myc and IRF4. Histone deacetylase inhibitors (HDACi) also downregulate c-Myc. We therefore determined whether IMiDs with HDACi trigger significant MM cell growth inhibition by inhibiting or downregulating c-Myc. Combination treatment of Len with non-selective HDACi suberoylanilide hydroxamic acid or class-I HDAC-selective inhibitor MS275 induces synergic cytotoxicity, associated with downregulation of c-Myc. Unexpectedly, we observed that decreased levels of cereblon (CRBN), a primary target protein of IMiDs, was triggered by these agents. Indeed, sequential treatment of MM cells with MS275 followed by Len shows less efficacy than simultaneous treatment with this combination. Importantly ACY1215, an HDAC6 inhibitor with minimal effects on class-I HDACs, together with Len induces synergistic MM cytotoxicity without alteration of CRBN expression. Our results showed that only modest class-I HDAC inhibition is able to induce synergistic MM cytotoxicity in combination with Len. These studies may provide the framework for utilizing HDACi in combination with Len to both avoid CRBN downregulation and enhance anti-MM activities.

## Introduction

Despite progress due to development of proteasome inhibitors (bortezomib, carfilzomib) and immunomodulatory drugs (IMiDs; thalidomide, lenalidomide, pomalidomide), novel combination treatment strategies are needed to further improve multiple myeloma (MM) patient outcome. Recent studies have shown cereblon to be a primary target of IMiDs:^1, 2, 3^ IMiDs bind to cereblon, an E3 ubiquitin ligase which facilitates ubiquitination of IKZF1 (Ikaros) and IKZF3 (Aiolos) followed by proteasomal degradation. Indeed, IMiDs downregulate IKZF1/3 within several hours, which is abrogated by proteasome inhibitors. Of note, knockdown of IKZF1/3 induces significant growth inhibition of MM cells.^4, 5^ Although human MM has multiple translocations involving IgH switch regions, c-MYC is infrequently involved as a partner in these translocations.^6^ However, it is commonly activated in MM, and knockdown of MYC induces MM cell death,^7^ suggesting that c-Myc represents a promising therapeutic target in MM. Importantly, it has also shown that IMiDs downregulate not only c-Myc, but also IRF4,^3, 8^ which has a central role in MM pathogenesis.^6^ These studies show that IMiDs inhibit multiple key molecules that mediate MM cell proliferation, survival and drug resistance in the context of the bone marrow (BM) microenvironment.

Histones are localized in the nucleus and, as the predominant protein components of chromatin, have a major role in modulating the binding of transcription factors to DNA. The activity of histones is regulated by their acetylation status, which is tightly mediated by both acetyltransferases and deacetylases. Histone deacetylases (HDACs) are divided into distinct classes: class-I (HDAC1, 2, 3 and 8), class-IIa (HDAC4, 5, 7 and 9), class-IIb (HDAC6 and 10), class-III (sirtuins) and class-IV (HDAC 11). HDACi are hydroxamic acids, benzamides, cyclic peptides, ketones or aliphatic acids, and each HDACi targets different isoforms of HDAC.^9^ Recent studies have shown that HDACi are promising anti-tumor agents in various malignancies and other diseases.^10^ In MM, we have shown that non-selective HDACi induce anti-MM activities in preclinical settings;^11, 12, 13^ however, their clinical activities are limited due to unfavorable toxicities including fatigue, diarrhea and thrombocytopenia attendant to broad inhibition of HDAC isoforms.^14^

To exploit anti-MM activities while minimizing toxicities of HDACi, class or isoform selective HDACi have recently been developed. For example, we have shown that HDAC6 selective inhibitors (tubacin, ACY1215) induce significant anti-MM activities in combination with proteasome inhibitors by blocking both proteasomal and aggresomal protein degradation,^15, 16, 17^ and early clinical trials demonstrate a favorable side effect profile.^18^ Most recently, we have developed HDAC3 selective small molecule inhibitor BG45, which also shows significant MM cell growth inhibition in an *in vivo* murine xenograft MM model.^19^ Since previous studies have shown that class-I/II HDAC inhibitors downregulate c-Myc expression,^20, 21^ we here examined whether various HDACi together with IMiDs trigger both downregulation of c-Myc and synergistic anti-MM activity, to provide the framework for combination clinical trials.

## Materials and methods

### Cell lines, patient MM cells and BM stromal cell

MM.1S and NCI-H929 cells were obtained from American Type Culture Collection (Manassas, VA, USA). All MM cell lines were cultured in RPMI-1640 (Gibco, Grand Island, NY, USA) containing 10% fetal bovine serum (Sigma Chemical Co., St. Louis, MO, USA), 2 μm l-glutamine, 100 U/ml penicillin and 100 μg/ml streptomycin (Gibco). Patient MM cells were purified as previously reported.^17^ To generate BM stromal cells (BMSCs), mononuclear cells separated by Ficoll-Hypaque density sedimentation from BM aspirates were cultured in RPMI-1640 containing 15% fetal bovine serum for 5–6 weeks. All experiments using patient samples were performed according to a protocol approved by the Institutional Review Board of Dana-Farber Cancer Institute.

### Reagents and antibodies

Suberoylanilide hydroxamic acid (SAHA, vorinostat), LBH589 (panobinostat), MS275 (entinostat), lenalidomide (Len), pomalidomide (Pom) and bortezomib (BTZ) were purchased from Selleck Chemicals (Houston, TX, USA). HDAC6 inhibitor ACY1215 (ricolinostat) was obtained from ChemieTek (Indianapolis, IN, USA). Anti-acetylated α-tubulin antibody (Ab) and -cereblon Abs were purchased from Sigma (St. Louis, MO, USA). Anti-c-Myc, anti-acetylated lysine, anti-glyceraldehyde 3-phosphate dehydrogenase (GAPDH), anti-caspase-8, anti-caspase-9, anti-cleaved-caspase-3, anti-poly (ADP-ribose) polymerase (PARP), anti-X-linked inhibitor of apoptosis protein (XIAP), Bcl2, anti-cIAP2, anti-α-tubulin, phospho-STAT3, HDAC6, IKZF1, IKZF3 and IRF4 Abs were obtained from Cell Signaling Technology (Danvers, MA, USA). Anti-IKZF1 Abs were purchased from Cell Signaling Technology or R&D Systems (Minneapolis, MN, USA) Figure 4d. Human interleukin-6, insulin-like growth factor 1, vascular endothelial growth factor and tumor necrosis factor α were obtained from R&D Systems.

### Cell growth assay

Cell growth was assessed by measuring 3-(4,5-dimethylthiazol-2-yl)-2,5-diphenyl tetrasodium bromide (MTT; Chemicon International, Temecula, CA, USA) dye absorbance. Cells were pulsed with 10 μl of 5 mg/ml MTT to each well for the last 4 h of 48 h and/or 72 h cultures, followed by 100 μl isopropanol containing 0.04 n HCl. Absorbance was measured at 570/630 nm using a spectrophotometer (Molecular Devices Corp., Sunnyvale, CA, USA).

### Immunoblotting

Cells cultured with the reagents were harvested, washed and lysed using RIPA (radioimmunoprecipitation assay) lysis buffer with 5 mm EDTA, 5 mm EGTA (ethylene glycol tetraacetic acid), 5 mm NaF, 1 mm Na_3_VO_4_, 1 mm PMSF (phenylmethanesulfonylfluoride), and complete protease inhibitor cocktail (Roche Diagnostics, Indianapolis, IN, USA). Whole-cell lysates were subjected to SDS-polyacrylamide gel electrophoresis, transferred to nitrocellulose membrane (Bio-Rad Laboratories, Hercules, CA, USA) and immunoblotted with specific Abs.

### Flow cytometric analysis

For annexin-V–PI staining, MM.1S cells cultured for 24 h with SAHA (0.5 μm) and/or lenalidomide (2.5 μm) were washed with phosphate-buffered saline and processed according to manufacturer's protocol (FITC Annexin V Apoptosis Detection Kit I; BD Pharmingen, San Diego, CA, USA).

### Co-cultures with BMSC culture media

To evaluate the effect of Len, ACY1215 or the combination on MM cell growth in the context of the BM microenvironment, MM.1S and H929 cells were cultured with these agents, in the presence or absence of BMSC culture supernatant (BMSC-CS). Cell growth was measured by MTT assay in triplicate or quadruplicate.

### siRNA transfection

For small interfering RNA (siRNA) transfection, scrambled, HDAC6 and cereblon (CRBN) ‘ON-TARGETplus SMARTpool siRNA' were purchased from Thermo Scientific (Lafayette, CO, USA). siRNA transfection was carried out by Amaxa electroporation system using ‘Cell Line Nucleofector Kit V' solution, according to manufacturer's protocol (Lonza, Koln, Germany).

### RNA extraction and reverse transcription PCR

RNA was extracted using Trizol (Invitrogen, Grand Island, NY, USA) and quantified by a Nanodrop spectrophotometer (Labtech, East Sussex, UK). Specifically, 5 × 10^6^ cells were pelleted, washed with cold phosphate-buffered saline and then resuspended in 1 ml trizol. They were then incubated with 1-bromo-3-chloropropane (Sigma), washed first with isopropyl alcohol and then with 75% ethanol, and resuspended in nuclease-free water (Invitrogen). After quantification, 2000 ng of RNA was used to synthesize cDNA via the Superscript II First strand synthesis Kit (Invitrogen), according to the manufacturer's instructions. To evaluate the expression levels of MYC, cereblon and GAPDH, quantitative real-time reverse transcription PCR (qRT-PCR) was performed using SYBR GREEN PCR Master Mix (Applied Biosystems, Life Technologies), after optimization of the primer conditions. cDNAs were diluted 1:100 or 1:1000 and amplified in a 20 μl reaction. Primers used (200 or 400 nm) are as follows: MYC, forward: TTTTTCGGGTAGTGGAAA, reverse: GCAGTAGAAATACGGCTGCAC; CRBN, forward: CAGTCTGCCGACATCACATAC, reverse: GCACCATACTGACTTCTTGAGGG; GAPDH forward: GAAGGTGAAGGTCGGAGTCA, reverse: GGGGTCATTGATGGCAACAATA. Thermal cycling conditions were: 10 min at 95 °C, 40 cycles at 95 °C for 15 s; followed by 1 min at 60 °C. qRT-PCR was performed on ABI Prism 7300 Sequence Detection System (Applied Biosystems). Data were analyzed using the delta Ct method, and GAPDH was used as an invariant control.

### Statistical analysis

Statistical significance of differences observed in drug-treated versus control cultures was determined using the Wilcoxon signed-ranks test. The minimal level of significance was *P*<0.05. The interaction between Len and HDAC inhibitors was analyzed by isobologram analysis using the CalcuSyn software program (Biosoft, Ferguson, MO, USA) to determine whether the combination was additive or synergistic; a combination index (CI)<1.0 indicates a synergistic effect.

## Results

### IMiDs and HDAC inhibitors downregulate c-Myc expression

**Figure 1:**
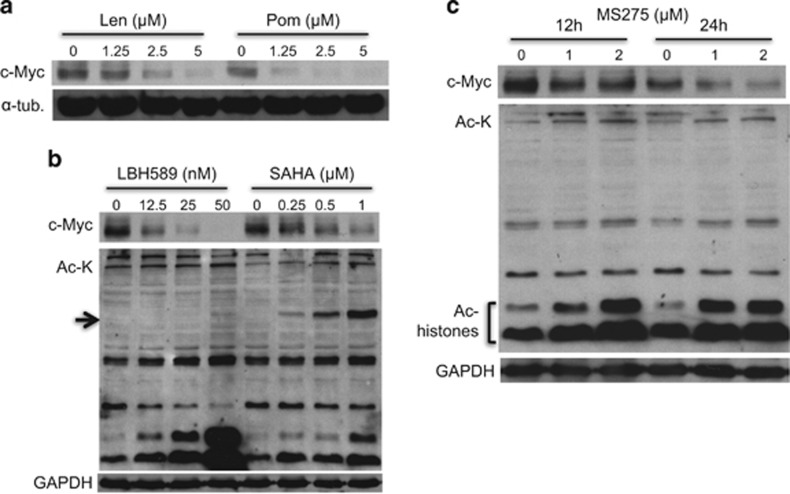
IMiDs and HDACi downregulate c-Myc. (**a**) MM.1S cells were cultured with Len or Pom for 48 h. (**b**) MM.1 S cells were cultured with LBH589 or SAHA for 48 h. (**c**) MM.1S cells were cultured with MS75 for 12 or 24 h. Whole-cell lysates were subjected to immunoblotting with indicated Abs. The arrow indicates acetylated-α-tubulin as a biomarker of HDAC6 inhibition.

c-Myc has a crucial role in MM pathogenesis, and previous studies have shown that IMiDs downregulate c-Myc expression in MM cells.^8^ We therefore first examined the inhibitory effect of Len and Pom on c-Myc expression in our setting. Both agents markedly downregulated c-Myc expression in MM.1S cells in a dose-dependent manner. Pom has even more potent inhibitory activity on c-Myc than Len (Figure 1a). HDAC inhibitors also have been shown to downregulate c-Myc in other cell types,^20, 21^ and we next examined their inhibitory effect on c-Myc expression in MM. In these experiments, we employed two class-I/II (LBH589, panobinostat; SAHA, vorinostat) and a class-I selective (MS275, entinostat) HDACi. Consistent with previous studies, LBH589 and SAHA downregulated c-Myc expression in a dose-dependent manner (Figure 1b). MS275 also significantly downregulated c-Myc expression associated with increased acetylation in histones, but not acetylation of the class-IIb HDAC6 substrate α-tubulin (Figure 1c). These results strongly suggest that downregulation of c-Myc by HDAC inhibitors is due to class-I HDAC inhibition.

### IMiDs in combination with HDAC inhibitors show synergistic cytotoxicity

**Figure 2:**
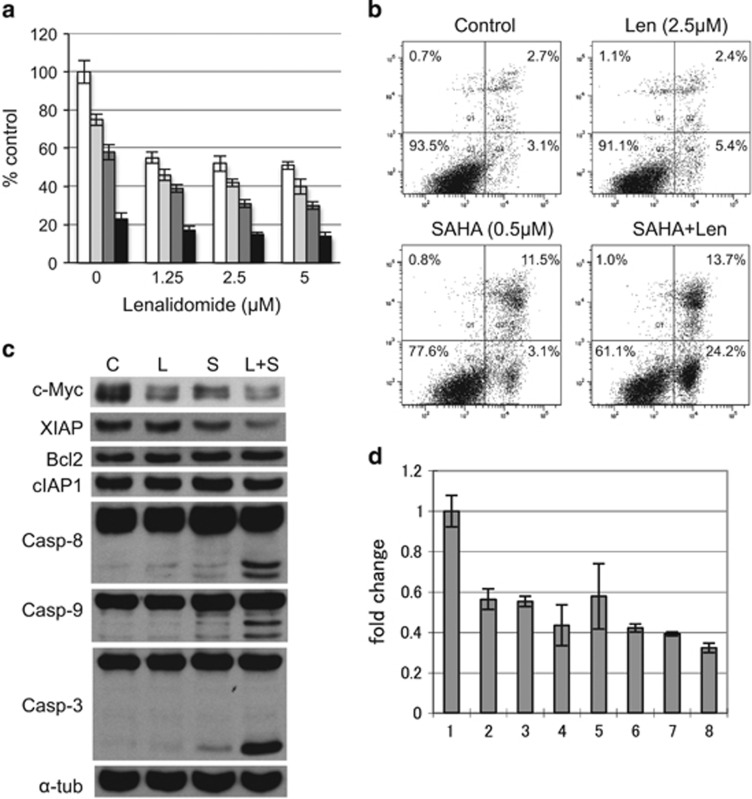
Len in combination with SAHA triggers synergistic cytotoxicity. (**a**) MM.1S cells were cultured with Len (1.25–5 μm ) with vehicle control (□), and with 0.25 (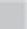), 0.5 (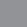) or 1 μm (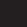) SAHA for 48 h. Cell growth was assessed by MTT assay, and data represent mean±s.d. from three independent experiments. CI was calculated by CalcuSyn software program. (**b**–**d**) MM.1S cells were cultured with Len (2.5 μm) in the presence or absence of SAHA (0.5 μm) for 24 h. (**b**) Cells were harvested and subjected to annexin-V (X-axis)/PI (Y-axis) staining. Numbers indicate cell population in each quadrant. (**c**) Whole-cell lysates were subjected to immunoblotting with indicated Abs. (**d**) Cells were subjected to qRT-PCR analysis of c-Myc; 1, control; 2, Len 1.25 μm; 3, Len 2.5 μm; 4, Len 5 μm; 5, SAHA 0.25 μm; 6, SAHA 0.5 μm; 7, Len 2.5 μm+SAHA 0.25 μm; 8, Len 2.5 μm+SAHA 0.5 μm. Fold changes were normalized with internal control (GAPDH).

We next examined the combination effect of IMiDs and HDACi on MM cytotoxicity. MTT assay showed that combination treatment induced synergistic cytoxicity in MM.1S cells (Figure 2a, Supplementary Figure S1). Annexin-V/PI staining confirmed that the combination triggered apoptosis. For example, the population of annexin-V-positive cells after treatment with Len or SAHA was 7.8% and 14.4%, respectively, which increased to 37.9% after combination treatment (Figure 2b). Immunoblotting was carried out to examine the molecular mechanism of apoptosis. Combination treatment markedly downregulated c-Myc and XIAP without affecting Bcl2 or cIAP1 anti-apoptotic proteins; as well as triggered cleavage of caspase-8, -9 and -3 (Figure 2c), indicating that Len with SAHA triggered apoptosis by activating caspases and downregulating anti-apoptotic factors. Of note, downregulation of c-Myc is due to inhibition of mRNA levels, evidenced by qRT-PCR (Figure 2d).

### HDACi can downregulate cereblon and antagonize the effect of IMiDs

**Figure 3:**
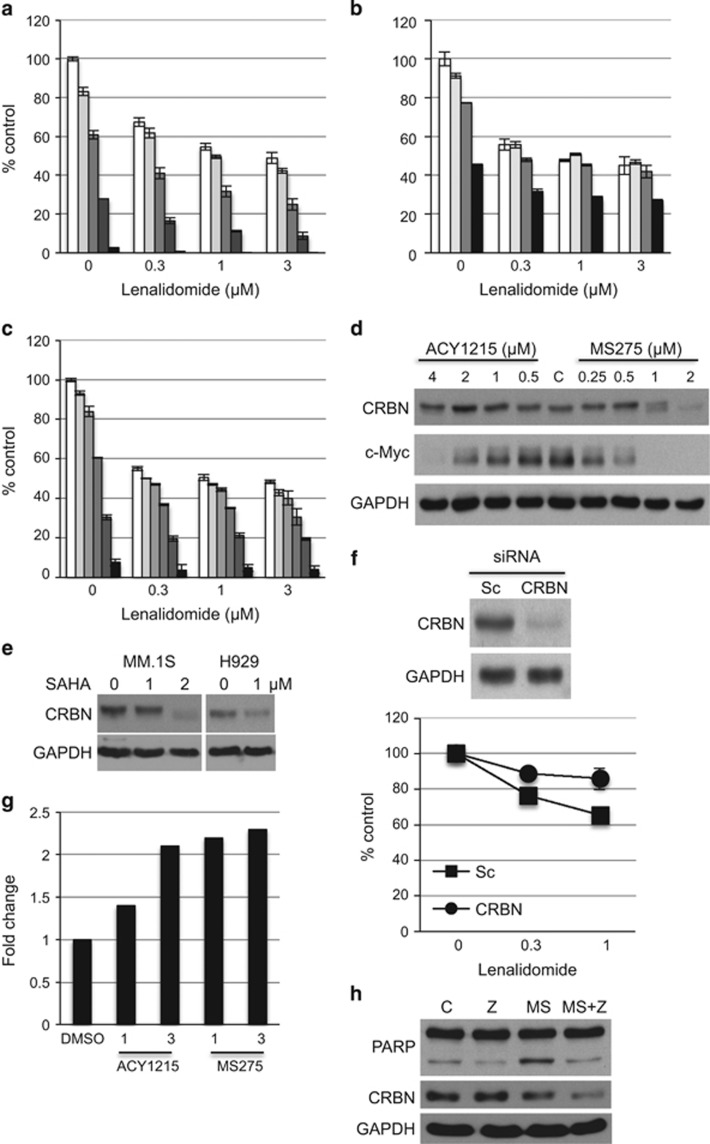
Class-I/II HDACi downregulate CRBN. (**a**) MM.1 S cells were simultaneously treated with Len in the presence of 0 (□), 0.125 (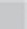), 0.25 (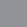), 0.5 (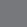) or 1 μm (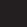) for 72 h. (**b**) MM.1S cells were treated with 0 (□), 0.031 (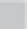), 0.062 (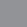) or 0.125 μm (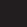) MS275 for 48 h. Cells were then further treated with Len (0.1, 0.3 and 1 μm) for 48 h. (**c**) MM.1S cells were treated with 0 (□), 0.125 (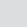), 0.25 (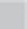), 0.5 (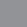), 1 (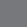) and 2 μm (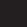) ACY1215 for 48 h. Cells were then further treated with Len (0.1, 0.3 and 1 μm) for 48 h. Cell growth was assessed by MTT assay. The data represent mean±s.d. from three independent experiments. CI was calculated by CalcuSyn software program. (**d**) MM.1S cells were cultured with increasing doses of ACY1215 or MS275 for 48 h. (**e**) MM.1S cells and H929 cells were treated with SAHA for 48 h. Whole-cell lysates were subjected to immunoblotting with indicated Abs. (**f**) H929 cells were transfected with scrambled (Sc) or CRBN-targeted siRNA. Whole-cell lysates were subjected to immunoblotting with indicated Abs (upper panel). Transfected cells were also cultured with Len (0.3 and 1 μm) for 72 h. Cell growth was assed by MTT assay. (**g**) MM.1S cells were treated with ACY1215 or MS275 (1 and 3 μm) for 48 h. mRNAs were subjected to qRT-PCR for CRBN. Fold changes were normalized with GAPDH. (**h**) MM.1S cells were cultured with MS275 (1 μm) in the absence or presence of Z-VAD-FMK (50 μm) for 24 h. Whole-cell lysates were subjected to immunoblotting with indicated Abs.

Synergistic cytotoxicity was also observed after Len treatment in combination with class-I HDACi MS275 (Figure 3a, Supplementary Figure S2) or ACY1215, an HDAC6 inhibitor with minimal class-I inhibitory effect, in MM.1S cells (Supplementary Figure S3); as well as in H929 cells treated with ACY1215 with Pom (Supplementary Figure S4). Since we have previously shown that sequential treatment of doxorubicin followed by bortezomib triggers significant cytotoxicity,^22^ we similarly examined whether pretreatment with MS275 enhances Len-induced cytotoxicity in MM.1S cells. Unexpectedly, we observed an antagonistic effect of MS275 on Len-induced cytotoxicity (Figure 3b, Supplementary Figure S5A). Importantly, this antagonistic effect against Len was not observed after pretreatment with ACY1215 on the same schedule (Figure 3c, Supplementary Figure S5B). Previous studies have shown that IMiDs bind to CRBN, followed by degradation of IKZF1/3;^4, 5^ moreover, downregulation of CRBN confers resistance to IMiDs treatment.^2, 3^ We therefore next examined whether various HDACi modulate expression and/or function of CRBN. Both MS275 and ACY1215 downregulated c-Myc in a dose-dependent manner; moreover, MS275 (>1 μm), but not ACY1215, also markedly suppressed CRBN expression (Figure 3d). Similar results were observed in MM.1S and H929 cells treated with SAHA with Len (Figure 3e). To examine whether downregulation of CRBN abrogates cytotoxicity of combination treatment, we knocked down CRBN using siRNA in H929 cells (Figure 3f, upper panel). Consistent with previous studies, we confirmed that CRBN knockdown cells acquired resistance to the Len treatment (Figure 3f, lower panel). Taken together, these results indicate that potent inhibition of class-I HDACs is not required for combination treatment with IMiDs to obtain synergistic cytotoxicity. Moreover, treatment with class-I/II or potent class-I HDACi before IMiD treatment antagonizes IMiD-induced cytotoxicty due to downregulation of CRBN.

Since HDACi downregulates c-Myc mRNA, we similarly examined mRNA level of *CRBN* after treatment with ACY1215 and MS275. Unlike c-Myc, mRNA of *CRBN* was upregulated by the treatment (Figure 3g), suggesting upregulation of mRNA due to a positive feedback mechanism. Since proteases (that is, caspases) cleave a number of proteins, we cultured MM cells with MS275 in the presence or absence of pan-caspase inhibitor Z-VAD-FMK (ZVAD). Although ZVAD inhibited MS275-induced cleavage of PARP and cytotoxicity, it did not block downregulation of cereblon (Figure 3h, Supplementary Figure S6), indicating a non-caspase-dependent mechanism of CRBN downregulation.

### ACY1215 downregulates IKZF1 and IKZF3

**Figure 4:**
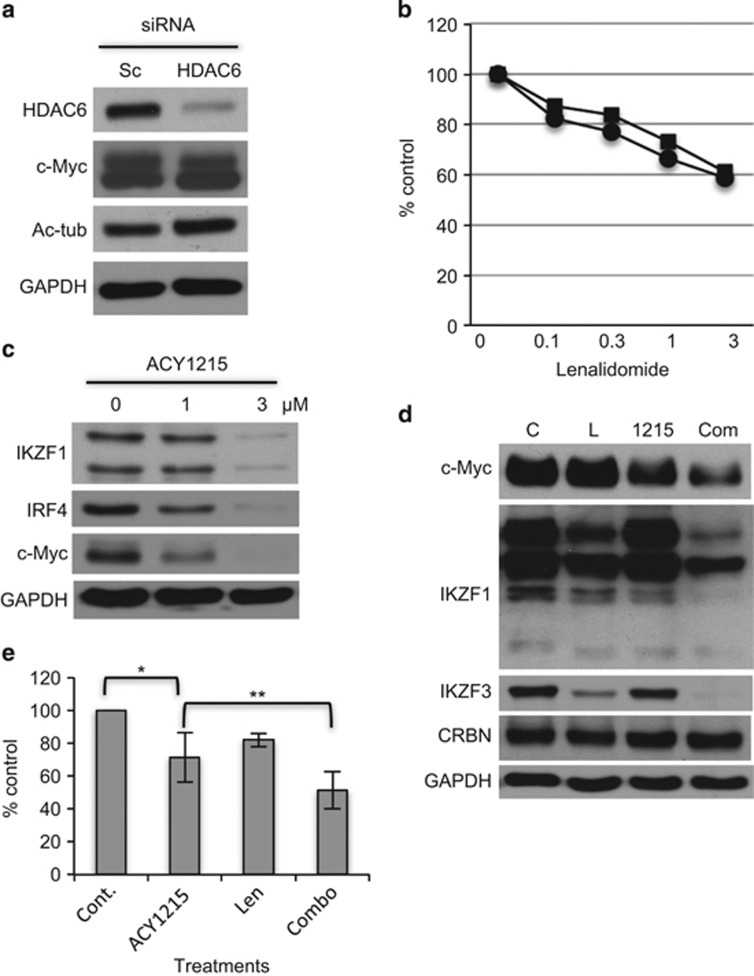
Len in combination with ACY1215 downregulates IKZF1/3. (**a**) H929 cells were transfected with scrambled (Sc) or HDAC6 targeted siRNA. Whole-cell lysates were subjected to immunoblotting with indicated Abs. (**b**) Transfected cells were also cultured with Len (0.3–3 μm) for 72 h. Cell growth was assessed by MTT assay. (**c**) MM.1S cells were cultured with Len (1 and 3 μm) for 48 h. Whole-cell lysates were subjected to immunoblotting. (**d**) MM.1S cells were cultured with ACY1215 (1 μm) for 16 h, and then treated with Len for 8 h (1 μm). Whole-cell lysates were subjected to immunoblotting with indicated Abs. (**e**) CD138-positive MM tumor cells from MM patients (*n*=7) were cultured with Len (2 μm) in the absence or presence of ACY1215 (2 μm) for 48 h. Cell growth was assessed by MTT assay. Data represent mean±s.d. from three independent experiments. *P<0.01; **P<0.02.

Since Len in combination with ACY1215-triggered synergistic cytotoxicity in MM cells (Figure 3), we next asked whether inhibition of HDAC6 mediated this effect. As in previous studies,^15^ we knocked down HDAC6 using targeted siRNA in H929 cells. HDAC6 knockdown did not alter c-Myc expression (Figure 4a), consistent with our experiments showing that c-Myc downregulation by HDAC inhibitors is due to class-I inhibitory effect (Figure 1). Moreover, HDAC6 knockdown did not enhance Len-induced cytotoxicity (Figure 4b), further indicating that this effect is not due to inhibition of class-IIb HDAC. These results indicate that HDAC6 does not modulate either c-Myc expression or sensitivity to Len treatment.

Since ACY1215 with Len triggered significant cytotoxicity without downregulating CRBN expression, we next examined the molecular mechanism mediating the synergistic effect of this combination treatment. IKZF1 (Ikaros) and IKZF3 (AIolos) have been shown to have crucial role in MM cell survival. Specifically, IKZF1/3 are degraded via activation of CRBN upon IMiD treatment, thereby triggering MM cell growth inhibition.^4, 5^ We here observed that ACY1215 markedly downregulated IKZF1, IRF4 and c-Myc in a dose-dependent manner (Figure 4c). Importantly, low doses of Len with ACY1215 significantly reduced expression of c-Myc, IKZF1 and IKZF3 without affecting CRBN expression, suggesting that downregulation of IKZF1/3 may contribute to the cytotoxicity induced by this combination. Len with ACY1215 treatment is similarly effective against primary MM cells from patients (*n*=7; Figure 4e).

### Len with ACY1215 blocks BMSC-induced upregulation of c-Myc

**Figure 5:**
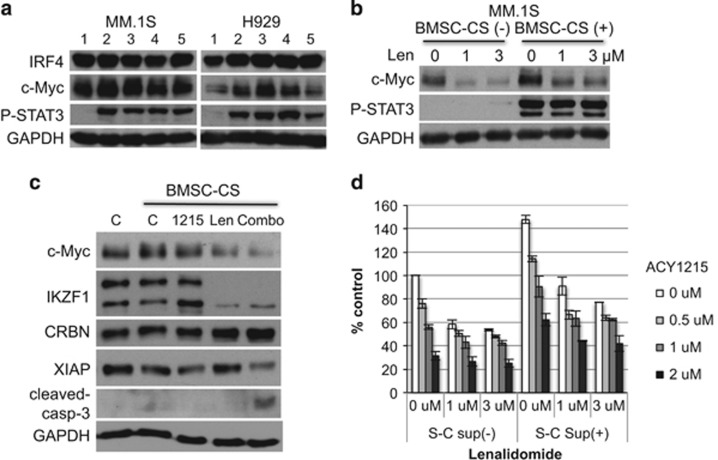
Len in combination with ACY1215 abrogates BMSC-induced c-Myc expression. (**a**) MM.1S and H929 cells were cultured for 48 h in the presence of BMSC-CS from five MM patients. (**b**) MM.1S cells were cultured for 48 h with BMSC-CS in the presence of Len. (**c**) MM.1S cells were cultured for 24 h with BMSC-CS in the presence of ACY1215 (2 μM), Len (1 μM) or both. Whole-cell lysates were subjected to immunoblotting with indicated Abs. (**d**) MM.1S cells were cultured for 48 h with BMSC-CS in the presence of ACY1215 and/or Len. Cell growth was assessed by MTT assay.

The BM microenvironment has a crucial role in MM pathogenesis by promoting tumor cell proliferation, survival and drug resistance.^23^ Therefore we next examined whether the BM microenvironment modulates c-Myc, IKZF1 or CRBN expression. BMSC-CS upregulated c-Myc, but not IRF4, expression in MM.1S and H929 cells (Figure 5a). Of note, phospho-STAT3 served as positive control for BMSC-SC-triggered signaling. Conversely, Len markedly downregulated c-Myc expression in a dose-dependent manner, even in the presence of BMSC-CS (Figure 5b). Importantly, combination treatment with Len and ACY1215 significantly downregulated c-Myc and XIAP, associated with caspase-3 cleavage (Figure 5c). Moreover, this combination induced synergistic MM cell growth inhibition even in the presence of BMAC-SC (Figure 5d). These data indicate that combination treatment can overcome BM microenvironment-mediated growth and drug resistance.

### ACY1215 enhances cytotoxicity induced by Len with dexamethasone or Len with bortezomib

**Figure 6:**
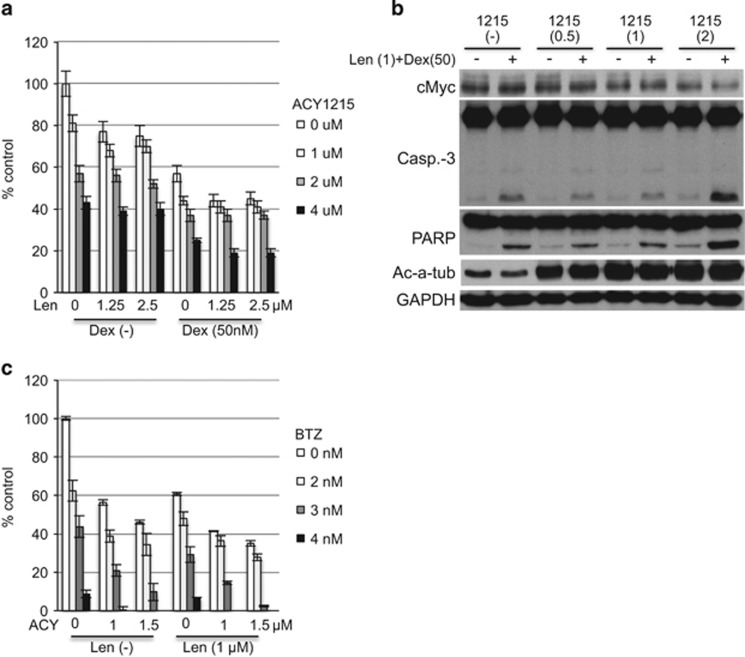
Len with ACY1215 in combination with Dex or BTZ induces significant cytotoxicity. (**a**) MM.1S cells were cultured for 48 h with Len (1.25 and 2.5 μm) and ACY1215 (1–4 μm) in the absence or presence of Dex (50 nm). Cell growth was assessed by MTT assay. (**b**) MM.1S cells were cultured for 24 h with Len (1 μm) and ACY1215 (1 and 2 μm) in the absence or presence of Dex (50 nm). Whole-cell lysates were subjected to immunoblotting with indicated Abs. (**c**) MM.1S cells were cultured for 48 h with Len (1 μm) and ACY1215 (1 and 1.5 μm) in the absence or presence of BTZ (2–4 nm). Data represent mean±s.d. from three independent experiments.

Since Len in combination with dexamethasone (Dex) is a standard treatment for MM, we next asked whether ACY1215 enhances cytotoxicity induced by this treatment. Indeed, ACY1215 enhanced MM cell growth inhibition by Len with Dex (Figure 6a). Immunoblotting demonstrated that ACY1215 downregulated c-Myc, as well as upregulated cleavage of caspase-3 and PARP, in a dose-dependent manner. Acetylated α-tubulin served as positive control for HDAC6 inhibition (Figure 6b).

Len with BTZ is another standard treatment option for MM. Moreover, we and others have show that HDAC inhibitors enhance BTZ-induced cytotoxicity. Specifically, HDAC6 inhibitor tubacin or ACY1215 with BTZ or carfilzomib show synergistic MM cytotoxicity, associated with accumulation of polyubiquitinated proteins and endoplasmic reticulum stress.^15, 16, 17^ Therefore, we also examined whether ACY1215 enhances cytotoxicity triggered by Len with BTZ. As shown in Figure 6c, ACY1215 in a dose-dependent manner enhanced growth inhibition triggered by Len with BTZ. Taken together, our results indicate that ACY1215 can be utilized to enhance MM cytotoxicity induced by standard MM treatment options that include Len and/or BTZ.

## Discussion

The proto-oncogene *c-MYC* encodes a transcription factor c-Myc, an oncoprotein which in turn is closely regulated by many mechanisms.^24^ In MM, *MYC* has a crucial role pathogenesis: expression of *MYC* is increased in newly diagnosed MM compared with monoclonal gammopathy of undetermined significance, suggesting that increased *MYC* expression is associated with progression from monoclonal gammopathy of undetermined significance to MM.^25, 26^ Moreover, c-Myc regulates its transcriptional target genes involved in MM cell proliferation, apoptosis and metabolism.^27^ Therefore, c-Myc is an attractive therapeutic target for the treatment of MM.

Len is a clinically active therapy that downregulates c-Myc expression in MM.^8^ In preclinical studies we have also reported that JQ1, a small molecule inhibitor of bromodomain (BRD) 4, can inhibit *MYC* RNA expression and MM cell growth.^28^ Moreover, synergistic anti-tumor activity of Len with another BRD inhibitor CPI203 has been reported in BTZ-resistant mantle cell lymphoma.^29^ Class-I/II HDACi SAHA and valproic acid also inhibit c-Myc expression in Ph1-positive acute leukemia^21^ and acute myeloid leukemia cells,^30^ respectively. Here we showed that IMiDs with HDACi trigger significant downregulation of c-Myc in MM, associated with synergistic cytotoxicity.

Several classes of HDACi targetting different isoforms^9^ are under clinical evaluation in MM. In this study, we first tested hydroxamic acid class HDACi SAHA and LBH589, as well as a benzamide class HDACi MS275. Consistent with previous studies,^21, 30^ each of these HDACi significantly downregulate c-Myc expression in MM cells. Importantly, SAHA and LBH589 inhibit both class-I and -II HDACs, whereas MS275 inhibits only class-I HDACs, suggesting that downregulation of c-Myc by these agents is due to inhibition of class-I HDACs (HDAC1, 2, 3 and 8). We further showed that these HDACi with Len show synergistic cytotoxicity associated with induction of caspase-8 and caspase-9 cleavage, activating both intrinsic and extrinsic apoptotic pathways. Previous studies have shown that XIAP has an important role in MM cell survival by inhibiting apoptosis.^31^ Interestingly, in our studies XIAP expression is markedly downregulated by Len with HDACi, indicating that combination treatment not only enhances MM cytotoxicity by activating apoptotic signaling, but also inhibits anti-apoptotic protein expression.

Recent studies have shown that CRBN is a direct binding protein of IMiDs^1, 3^ and act as an E3 ligase of IKZF1 and IKZF3. More specifically, IMiDs bind to cereblon and promote proteasomal degradation of IKZF1 and IKZF3 to trigger MM cell growth inhibition.^4, 5^ Previous studies show that knockdown of CRBN confers resistance to IMiDs treatment.^2, 3^ In this study, we confirmed that CRBN knockdown H929 cells are less sensitive to IMiD treatment than parental cells. Importantly, we found that class-I/II HDACi (SAHA) and class-I-selective HDACi (MS275)-downregulated CRBN in a caspase-independent manner. Consistent with this result, sequential treatment with MS275 followed by Len was antagonistic, whereas simultaneous treatment triggers synergistic cytotoxicity. Previous studies in a murine xenograft model have shown efficacy of combination treatment of Len with LBH589, and that mice receiving combination treatment had longer survival than those treated with either Len or LBH589 alone.^32^ Importantly, our results suggest that potent class-I/II or class-I-selective HDACi reduce MM cell sensitivity to IMiDs due to downregulation of CRBN. Therefore HDACi belonging to different classes and/or targeting different HDAC isoforms, as well as modification of treatment schedule, may be useful to maximize anti-MM activities of Len-HDACi combination therapies.

ACY1215 is the first-in-class clinically relevant HDAC6i with minimal blockade of class-I HDACs. ACY1215 in combination with proteasome inhibitors shows synergistic cytotoxicity in MM cells,^16, 17^ and combination treatment of ACY1215 with Len or BTZ is under clinical evaluation.^33^ Interestingly and in contrast to MS275 with Len combination, we did not observe downregulation of CRBN when ACY1215 was added to Len. In addition, HDAC6 knockdown was not able to promote enhanced cytotoxicity induced by Len. These data suggest that modest class-I inhibitory activity of ACY1215, rather than its class-IIb HDAC inhibitory activity, is mediating the enhanced cytotoxicity of this combination therapy. Taken together, these results have important clinical implications and suggest that only modest inhibition of class-I HDACs is required to induce synergistic cytotoxicity with Len, and that more potent inhibition of class-I HDACs triggers downregulation of CRBN, thereby compromising the efficacy of combination therapy.

Having shown synergistic MM cytotoxicity triggered by Len and ACY1215 without altering CRBN expression, we further examined mechanisms mediating cytotoxicity of this combination treatment. IKZF1 and IKZF3 are major targets of IMiDs downstream of cereblon, associated with MM cell growth inhibition. Recently, >200 CRBN binding proteins were identified which changed their expression after Len treatment.^34^ We here recognized that ACY1215 monotherapy downregulated IKZF1 only at relatively high doses; however, it markedly inhibited both IKZF1 and IKZF3 at low doses when combined with Len. Moreover, in this study all eight IKZF1 isoforms^35^ were downregulated by the combination of ACY1215 Len treatment, without affecting CRBN expression. Ongoing studies are further delineating molecular mechanisms of this combination effect.

The BM microenvironment has a crucial role in proliferation, survival and drug resistance of MM cells.^23^ Specifically, cell proliferation and drug resistance are mediated by soluble factors including interleukin-6, insulin-like growth factor 1 and vascular endothelial growth factor. We therefore determined whether combination treatment of Len with ACY1215 overcomes survival/anti-apoptotic signaling triggered by these factors. To avoid contamination of BMSCs in immunoblotting experiments, we cultured MM cells with conditioned medium harvested from BMSCs, which has been well validated in our previous studies.^36, 37^ We observed marked upregulation of c-Myc induced by BMSC-CS. Importantly, our results therefore suggest that the BM microenvironment upregulates c-Myc, thereby promoting MM cell proliferation and survival; conversely, Len with ACY1215 combination treatment can downregulate c-Myc and mediate MM cytotoxicity even in the BM milieu.

Three-drug combination regimens incorporating corticosteroids are common treatment options in MM. For example, RVD (Revlimid+Velcade+Dexamethasone) is a one of the most effective combination treatment strategies for relapsed/refractory MM^38^ and newly diagnosed MM,^39^ as well as for maintenance therapy in MM.^40^ We therefore further examined combination treatment of Len plus ACY1215 with or without Dex, and observed that Dex further enhanced cytotoxicity and apoptosis. We and others have shown that HDAC6 inhibition synergistically enhances cytotoxicity of BTZ^15, 16^ or carfilzomib^17^ in MM and non-Hodgkin lymphoma cells^41^ by blocking protein degradation via both the aggresomal and protesomal pathways. As expected, BTZ significantly augmented cytotoxicity induced by Len-Dex-ACY1215 combination treatment. Taken together, our results show that ACY1215 may enhance anti-MM activities of Len/Dex in combination BTZ due to its inhibitory activities against class-I and class-IIb HDAC, respectively, providing the preclinical rationale for RVD ACY1215 clinical trials to further improve patient outcome.

In conclusion, our results demonstrate that IMiDs with HDACi induce synergistic cytotoxicity in MM, associated with downregulation of c-Myc. Importantly, choice of HDACi and treatment schedules should be optimized to enhance cytotoxicity without downregulating CRBN expression. In particular potent broad class-I/II HDACi can downregulate CRBN and antagonize Len; in contrast more selective HDACi with modest class-I HDAC inhibitory activity of ACY1215 does not downregulate CRBN, thereby allowing for synergistic MM cytotoxicity.

## Notes

TH is a consultant for Acetylon Pharmaceuticals. KCA is a member of advisory board for Celgene, Millennium, Gilead, Bristol-Myers Squibb and Sanofi-Aventis, and is a scientific founder and member of advisory board of Acetylon and Oncopep. Y-TT is a consultant for Onyx. PGR is a member of advisory board for Celgene, Millennium, Johnson & Johnson, Novartis and Bristol-Myers Squibb. NCM is a member of advisory board for Millennium, Celgene, and Novartis. The remaining authors declare no conflicts of interests.
