# Supplementary material for: Rational combination treatment with histone deacetylase inhibitors and immunomodulatory drugs in multiple myeloma
Source: Blood Cancer J. 2015 May 15;5(5):e312–. doi: 10.1038/bcj.2015.38 (PMC4476017; doi:10.1038/bcj.2015.38)
Supplement: Supplementary Information [file bcj201538x1.doc]

**Supplementary figure legends**

**Figure S1. Simultaneous treatment of Len with SAHA shows synergistic cytotoxicity in MM.1S cells.**

MM.1S cells were simultaneously treated with Len in combination with SAHA (Figure 2A). Combination index (CI) was calculated by CalcuSyn software program.

**Figure S2.** **Simultaneous treatment of Len with SAHA shows synergistic cytotoxicity in MM.1S cells.**

MM.1S cells were simultaneously treated with Len in combination with MS275 (Figure 3A). Combination index (CI) was calculated by CalcuSyn software program.

**Figure S3.** **Simultaneous treatment of Len with ACY1215 shows synergistic cytotoxicity in MM.1S cells.**

(A) MM.1S cells were simultaneously treated with Len (0.1 - 1 μM) in combination with ACY1215 (0.25 - 4 μM) for 72h. Cell growth was assessed by MTT assay. Data represent mean ± SD from average of 3 independent experiments. (B) Combination index (CI) was calculated by CalcuSyn software program.

**Figure S4.** **Sequential** **treatment of ACY1215 with shows synergistic cytotoxicity in H929 cells.**

(A) H929 cells were treated with ACY1215 for 48h, then treated with Pom for additional 48h. Data represent mean ± SD from average of 3 independent experiments. (B) Combination index (CI) was calculated by CalcuSyn software program.

**Figure S5.** **Sequential** **treatment of MS275 with Len, but not ACY1215 with Len, shows antagonistic cytotoxicity in MM.1S cells.**

(A) MM.1S cells were sequentially treated with MS275 followed by Len (Figure 3B). (B) MM.1S cells were sequentially treated with ACY1215 followed by Len (Figure 3C). Combination index (CI) was calculated by CalcuSyn software program.

**Figure S6. Z-VAD-FMK blocks MS275-induced cytotoxicity.**

MM.1S cells were cultured with MS275 (1 μM) in the absence or presence (50 μM) of Z-VAD-FMK for 24h. Cell viability was assessed by trypan-blue test.
