# Supplementary figures and images for: Rational combination treatment with histone deacetylase inhibitors and immunomodulatory drugs in multiple myeloma
Source: Blood Cancer J. 2015 May 15;5(5):e312–. doi: 10.1038/bcj.2015.38 (PMC4476017; doi:10.1038/bcj.2015.38)

MM.1S+SAHA+Len (72h, simultaneous)

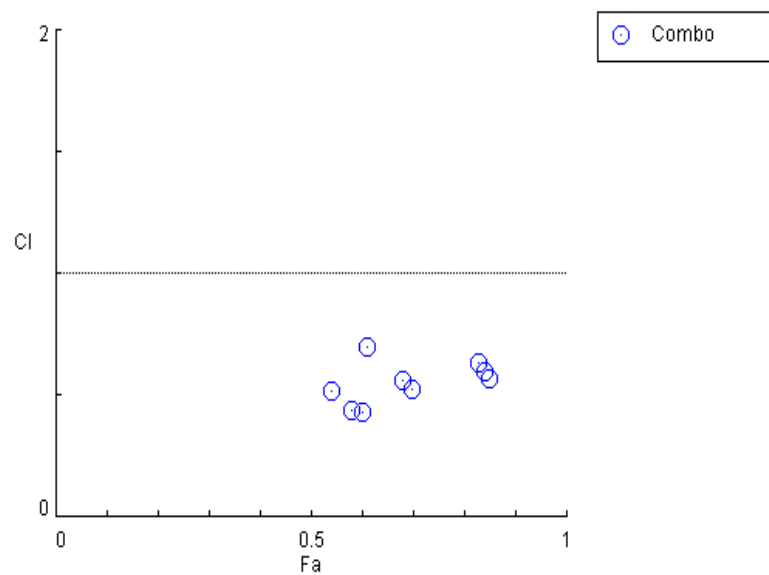

Suppl. Figure S1

Supplement: Supplementary Figure 1 [file bcj201538x2.pdf]

MM.1S + Len + MS275 (72h, simultaneous)

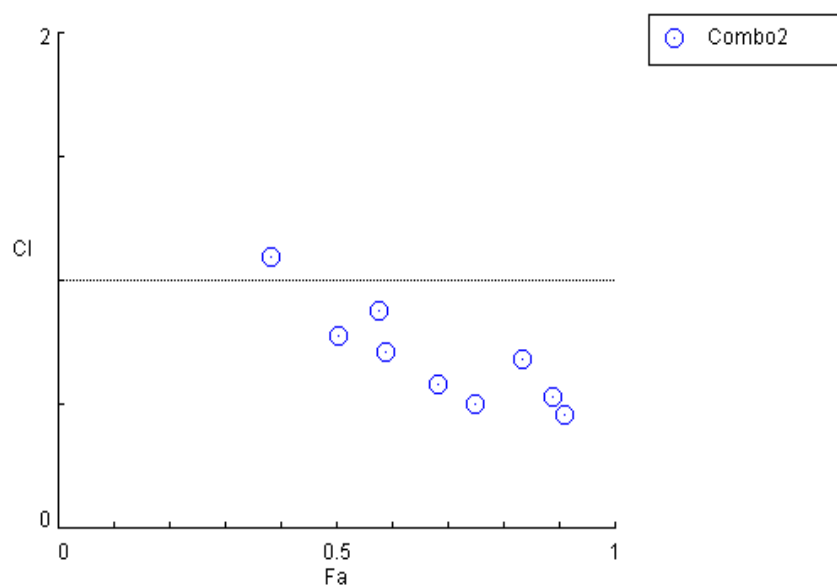

Suppl. Figure S2

Supplement: Supplementary Figure 2 [file bcj201538x3.pdf]

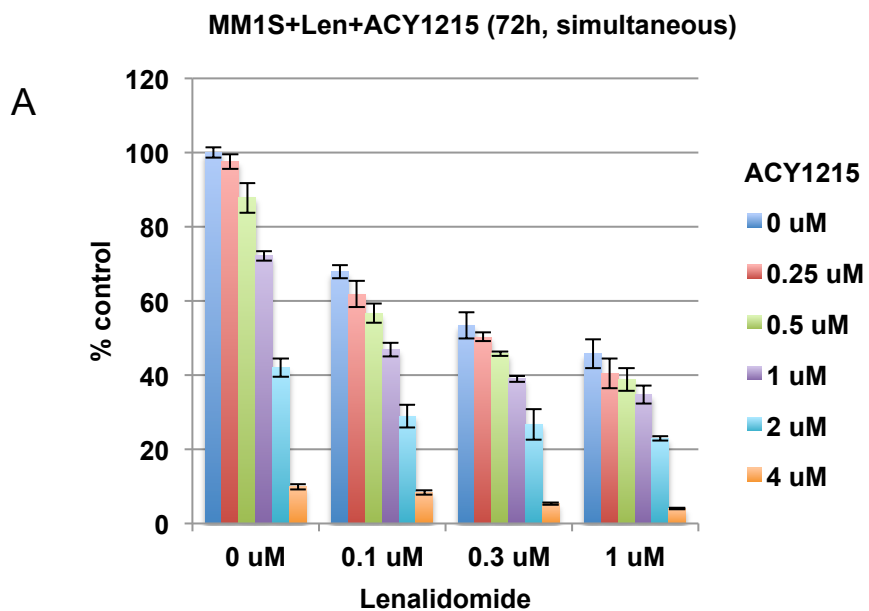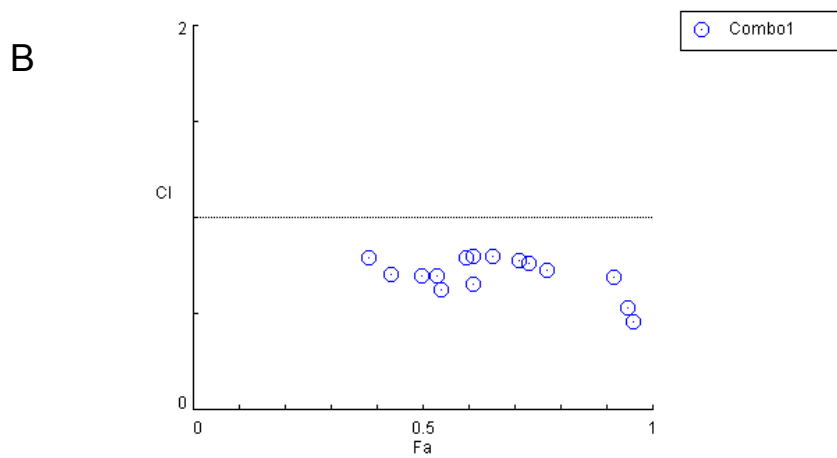

Suppl. Figure S3

Supplement: Supplementary Figure 3 [file bcj201538x4.pdf]

# H929 + Pom (72h, simultaneous)

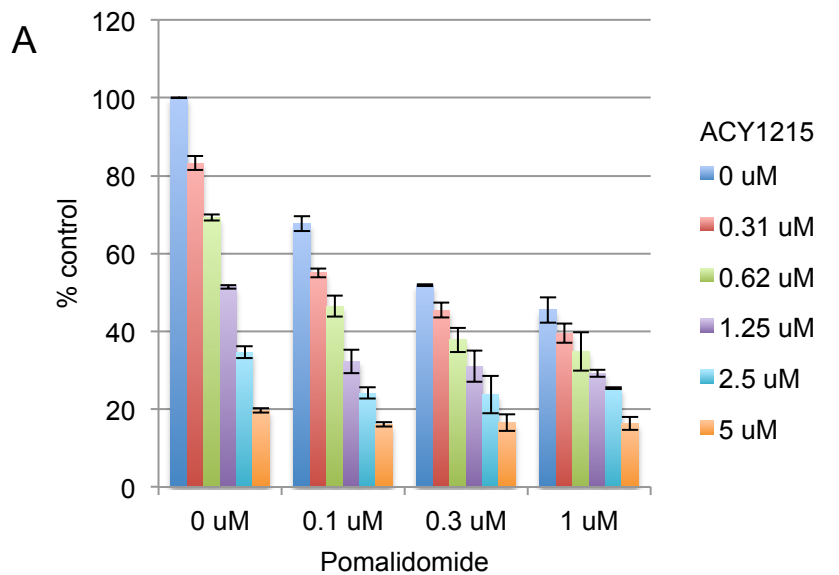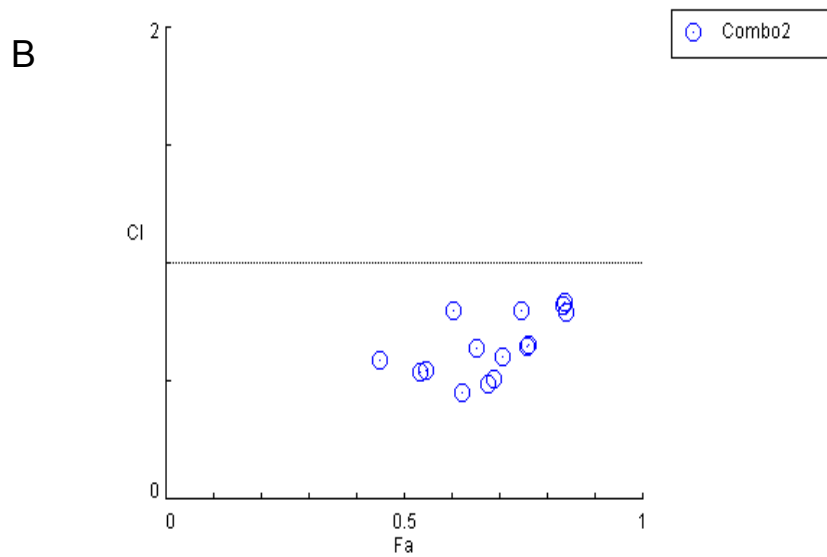

Suppl. Figure S4

Supplement: Supplementary Figure 4 [file bcj201538x5.pdf]

MM.1S + MS275 + Len (MS275 48h, then Len 48h)

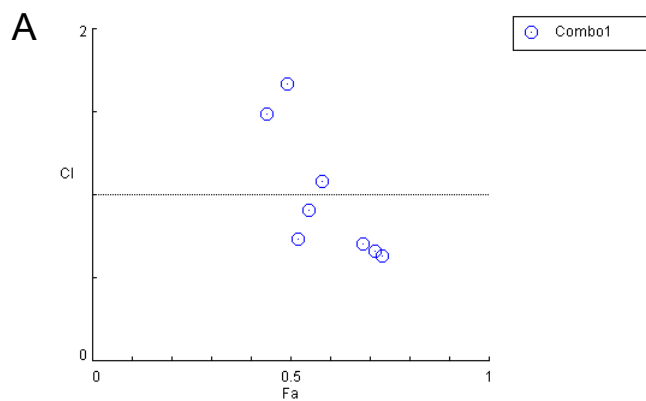

MM.1S + ACY1215 + Len (ACY1215 48h, then Len 48h)

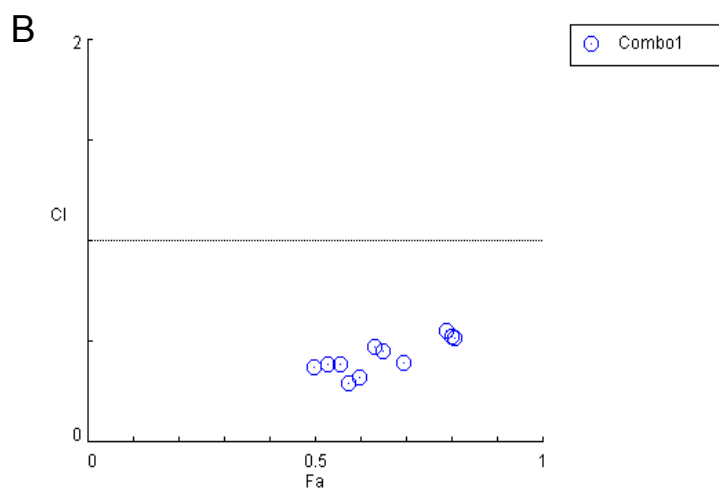

Suppl. Figure S5

Supplement: Supplementary Figure 5 [file bcj201538x6.pdf]

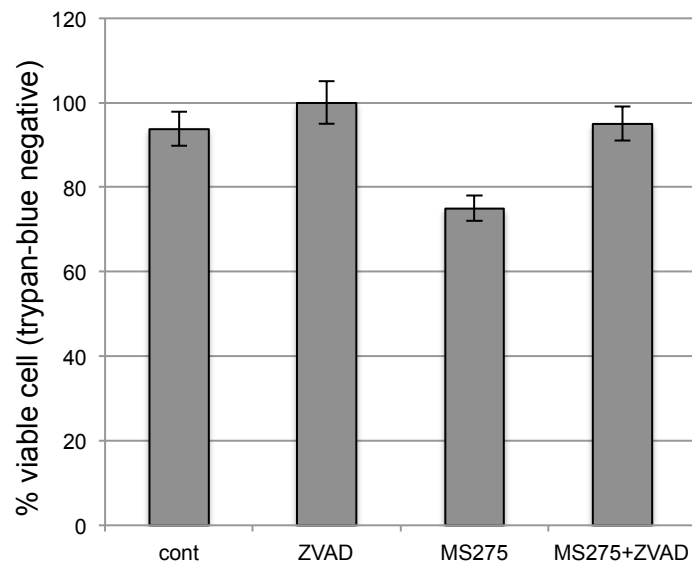

Suppl. Figure S6

Supplement: Supplementary Figure 6 [file bcj201538x7.pdf]
